# Response to global public health emergency: Overview and lessons from Chinese experience against COVID-19

**DOI:** 10.7189/jogh.13.03016

**Published:** 2023-03-31

**Authors:** Yuxuan Yang, Min Yu

**Affiliations:** Department of Health Service Support Research, Academy of Military Medical Sciences, Beijing, China

Coronavirus 2019 (COVID-19) has brought people’s attention to countries’ public health emergency preparedness and response (PHEPR). In the WHO strategic framework, governance, capacities, and resources are the core components for effective PHEPR [1]. China, the first country to encounter COVID-19, has gained a lot of experience in PHEPR during the past three years. We gathered data from published reports, the National Health Commission (NHC), WHO websites, and news releases, aiming to document and draw lessons from China’s response to COVID-19 by stages based on the PHEPR framework.

## EMERGENCY STAGE

The emergency stage refers to the period of the first wave of virus transmission, starting from the detection of the first case in Wuhan. It lasted for six months from December 2019 to April 2020.

### Governance

Both local and central governments at this stage reacted promptly to the appearance of an unknown disease. The NHC issued an urgent notice on unknown pneumonia within three days after the first case had been detected, followed by nine documents on prevention, control, and treatment protocol for unknown pneumonia within five days [2]. During this stage, the NHC updated the Protocol on Prevention and Control of COVID-19 seven times as knowledge about the virus increased [3]. The goal was to clear all the cases and prevent any new infections through the “Zero Covid-19 Case Policy” at any cost [4]. Therefore, four days after confirming that COVID-19 spread among people, the central government decided to lock down Wuhan, the epicentre of the outbreak, followed by other cities, to prevent further transmission [2]. The 76-day lockdown prevented more than 710 000 people outside Wuhan from being infected domestically, reducing the number of potential infections by 96% and delaying the spread of the virus to other cities by 2.91 days [5].

### Capacity

The ability to identify, detect, and treat COVID-19 was critical at the beginning of the outbreak. China CDC isolated the first novel coronavirus strain successfully within 11 days of the first case, which the WHO praised as a notable achievement [2]. In terms of detection, the initial version of nucleic acid test kits was developed within 15 days after the first case. Due to the limited nucleic antigen testing (NAT) capacity, tests were only provided to patients with fever and close contacts of confirmed cases. Regarding treatment, both western and Chinese medicine were used to treat COVID-19 patients. Starting from the 3^rd^ protocol, Chinese medicine was used to treat patients with mild symptoms and proved to be effective in preventing severe conditions [6,7].

### Resource

To address the scarcity of medical supplies while patient numbers surged, the central medical supply support group first directed most supplies to Wuhan. Simultaneously, the government encouraged relevant manufacturers to switch their product lines towards producing medical supplies temporarily, while also procuring them internationally. To counter the lack of hospital beds, two makeshift hospitals with 2600 beds were built in 12 days [8]. The government set up over 15 000 fever clinics throughout the country and appointed over 2000 hospitals to treat COVID-19 patients [9]. However, the lack of healthcare workers was challenging, and the government directed both military and civilian medics all over the country to Wuhan to treat patients and perform epidemiological investigations [10].

## ROUTINE COVID-19 CONTROL STAGE

The routine COVID-19 control stage refers to the period after the first wave of COVID-19, until Delta and Omicron variants entered China. It lasted for 13 months from April 2020 to May 2021. At this stage, the activities focused on containing the disease and minimising its influence on normal life and production.

### Governance

The focus of disease containment shifted from “preventing the coronavirus from spreading within the country or beyond” to “preventing imported infection from causing a new wave of the epidemic” [2]. The zero COVID-19 case policy was still in effect. The whole-city lockdowns were scaled based on the risk level (low, medium, or high) of a basic area, which was normally one subdistrict or one village [11]. Lockdowns were issued to medium and high-risk areas. Since precise and differentiated epidemic control strategies were encouraged, local governments had the authority to set their risk identification standards under the central CDC’s supervision.

**Figure:**
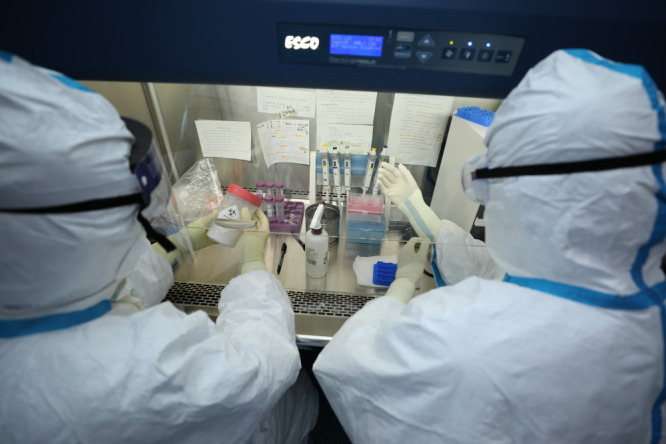
Photo: Medical workers from Tianjin Hedong CDC conducting nucleic acid test. Source: Li R (2020), used with permission.

### Capacity

Disease prevention technologies were important at this stage. Big data technology was first used in epidemiological investigations during the COVID-19 pandemic. With the users’ permission, telecommunication operators could locate them based on mobile phone signalling [12]. Health QR codes linked to people’s travel history, health conditions, NAT results, and vaccination status, were introduced to help contain the pandemic [12]. People were required to present their Health QR code whenever they entered any public place. Epidemiologists used these data to sort out the life trajectories of infected people, track the history of exposure to the crowd, and locate the source of infection and close contact groups. Big data technology helped with quicker and more precise detection of infected individuals.

### Resource

While medical supplies were no longer scarce, the lack of space for close-contact quarantine was sometimes challenging. Some shelter hospitals (temporary hospitals built by converting existing public venues) and civilian hotels were taken over as quarantine spaces. Since only regional outbreaks happened occasionally, the demand gap for healthcare workers decreased and only civilian medics travelled around the country to offer support at epicentres.

## DYNAMIC ZERO COVID-19 CASE STAGE

The dynamic zero COVID-19 case stage refers to the period from the Delta variant entering China to November 2022. As the virus mutated, the battle against COVID-19 seemed to have protracted. When the virus became less deadly and more transmissible, the disease containment strategy focused on “coexisting with COVID-19”.

### Governance

After the Delta and Omicron variants entered China, the Chinese government realized it could not achieve the goal of “zero cases” and changed its policy to “Dynamic Zero COVID-19 Policy” [13]. The “Dynamic Zero COVID-19 Policy” prioritizes the detection of infections and possible close contacts as fast and precisely as possible. Region-wide static management, which allows for activities that ensure the livelihood of citizens (e.g., those at hospitals and markets), replaced the lockdown strategy that caused massive standstills of everyday activities. The whole-of-population NAT strategy was adopted and citywide regular NATs were promoted by local governments. This strategy, alongside region-wide static management, played an important role at this stage. For example, Shangai did not adopt either approach against the Omicron variant as Tianjin did, so its confirmed case number rose to 53.13 times that of Tianjin (Shanghai's population is 1.79 times that of Tianjin) [14,15].

### Capacity

Boosting NAT capacity and expanding the scope of testing became the main target at this stage. In metropolises like Beijing, people had to present negative NAT results from the last 72 hours to enter public places. To ensure full coverage and availability of NATs and avoid the financial burden on people, some local governments covered their costs. As of April 2022, there were 13 100 medical institutions equipped with testing capabilities, with a testing capacity of 51.65 million tubes per day [16]. Vaccination was also important at this stage. The government paid for all vaccinations and encouraged people (excluding elders and children) to get vaccinated. The vaccination rate in China reached 90.74% at the end of March 2022 [17].

### Resource

Shelter hospitals were constructed to treat the increasing number of patients presenting with mild symptoms. This approach was strategically successful in reducing infection and transmission in communities and preventing the worsening of mild cases. Simultaneously, a lack of manpower occasionally posed a challenge, and more manpower was reserved for conducting NATs.

## LOOKING FORWARD

On 7 December 2022, the Chinese government released a circular on further optimizing the COVID-19 response, aiming to provide guidelines on precise disease containment strategies to both protect the people’s health and minimize the pandemic’s impact on economic and social development [18].

The whole-of-population NAT strategy was no longer encouraged; people did not need to present negative NAT results or health codes when entering public places (excluding schools, nursing homes, and other places with a large number of vulnerable populations); lockdowns were no longer issued and risk areas were downsized to buildings, floors, and even households. China expected the number of confirmed cases and severe cases to rise, putting significant pressure on its health system and challenging its resilience [18]. Therefore, the focus of governance, capacity, and resource shifted from prevention to treatment.

As of 14 January 2023, a month after the optimization, a total of 59 983 COVID-19-related deaths had been reported [19]. The shift from a disease containment strategy was not a dismissal of previous strategies, but a regular shift toward a scientific disease control measure as the pandemic developed. According to Dr Zunyou Wu, the Chief Expert of Epidemiology of China CDC, three years of strict COVID-19 containment strategy were critical for protecting people’s lives, since the occurrence of severe cases dropped from 16.47% in 2020 to 0.18% in 2022 and the death rate dropped from 5.18% to 0.04% [20]. As virulence decreased, three years of strict disease control and prevention gave China the chance to expand its vaccination rate, build capacity to deal with a large number of confirmed cases, and optimize its health system for COVID-19 control measures.

## LESSONS LEARNT FROM CHINA’S EXPERIENCE DURING THIS PANDEMIC

As the pandemic changes, policies and strategies to contain it should also be dynamic and flexible. Countries around the world may understand “coexisting with COVID-19” differently and have different opinions on COVID-19 governance. Many countries have tried different lockdown strategies and hold different perspectives on their effectiveness and rationality. For developing countries with high population density, a large vulnerable population (elders and children), and limited medical resources, the “Dynamic Zero Covid-19 Policy” seems to be a good choice from both health and economic perspectives. As the vaccination rate increases and the mortality rate decreases, containment strategies may become less strict, but countries should not relax their vigilance until the pandemic is over.

Different pandemic stages necessitate effective and reasonable capacity and resource planning to adapt to the changing circumstances, especially if resources are limited. Notably, a lack of manpower is unavoidable and challenges a country's overall PHEPR. Therefore, the ability to mobilize healthcare workers around the country is critical. Due to system differences from western countries, the Chinese central government has full control over all resources and manpower, ensuring that any COVID-19 policy can eventually be fully executed.

Containing a global public health emergency is a complicated task that requires joint societal and global efforts. From the Chinese experience, people can understand the importance of cooperation between the government and its people, between central authority and local authorities, between military and civilians, and among different industries. Every citizen is responsible for disease containment, and no strategies will work until all the people are fully committed to their responsibilities. The same philosophy works for the international community. Countries should be aware that no country is safe until every country is safe.
